# Effect of Treatment of Obstructive Sleep Apnea by Uvulopalatoplasty on Seizure Outcomes: A Case Report

**Published:** 2017-11

**Authors:** Gholamreza Shirani, Mahnaz Arshad, Xaniar Mahmoudi, Sergic Azarians

**Affiliations:** 1 Assistant Professor, Dental Research Center, Dentistry Research Institute, Tehran University of Medical Sciences, Tehran, Iran; Department of Oral and Maxillofacial Surgery, School of Dentistry, Tehran University of Medical Sciences, Tehran, Iran; 2 Assistant Professor, Dental Research Center, Dentistry Research Institute, Tehran University of Medical Sciences, Tehran, Iran; Department of Prosthodontics, School of Dentistry, International Campus, Tehran University of Medical Sciences, Tehran, Iran; 3 Dental Student, School of Dentistry, International Campus, Tehran University of Medical Sciences, Tehran, Iran; 4 Neurologist, Private Practice, Tehran, Iran

**Keywords:** Sleep Apnea, Epilepsy, Seizure

## Abstract

It is estimated that one-third of the people with refractory epilepsy suffer from obstructive sleep apnea (OSA). In a patient presenting with OSA symptoms and epilepsy, removing a portion of the soft palate (uvulopalatoplasty) can be considered the treatment of choice for eliminating the OSA and decreasing the seizures.

Here, we report the results of our surgical approach by which the patient’s problems completely resolved, and we observed no symptoms of OSA or epilepsy after the surgery. After 10 years of follow-up, the patient is seizure-free and does not need any antiepileptic drugs.

## INTRODUCTION

An epileptic seizure is an episode of unusual and uncontrollable motor, intuitive, or psychological behavior caused by reduplicated, hypersynchronous electrochemical activity that emanates from the cerebrum [1]. Epilepsy can occur as a result of a mutation in the gene that controls the neural behaviors. It may cause major brain damage, stroke, infection, and malignant tumors [2]. It has been estimated that more than 50 million people worldwide are likely to have epilepsy [3]. More than one-third of the epileptic patients have obstructive sleep apnea (OSA), and this illness is more prevalent among the patients with medically refractory epilepsy [4,5]. OSA is caused by partial or complete blockage of the airways during sleep. The obstruction in the pharyngeal area results in loud snoring, hypoxia and repeated apnea; as a result, the patients have an unrestful fragmented sleep and inordinate daytime sleepiness. The disorder is associated with hypertension, cardiovascular disease, impotence and emotional problems [4,5].

Evidence shows that about 4% of women and 6% of men suffer from OSA [5]. Many studies have proven that OSA is associated with major disorders of the nervous system, memory, and learning and executive function of the brain. However, the main cause of the disease is still unknown [6]. Neural imaging allows a more precise study of the brain function and neural changes [6].

Nonsurgical remedial choices for OSA include weight loss, a correct sleeping position, continuous positive airway pressure (CPAP) therapy, avoiding alcohol and other depressants of the central nervous system (CNS), certain dental appliances and surgical methods [7]. Several soft-tissue surgical techniques are available for the treatment of OSA such as uvulopalatopharyngoplasty (UPPP), radiofrequency ablation, maxillomandibular advancement, genioplasty and tracheostomy [8]. Maxillomandibular advancement surgery is the best treatment option for the patients who are reluctant to use CPAP therapy or dental appliances [7–9]. UPPP is a surgical method widely used for the treatment of OSA [9].

A study of 269 patients treated with UPPP showed that the success rate of this surgery is 59% with a simultaneous tonsillectomy and 30% without tonsillectomy. The long-term success rate was 60.5% after 3 to 12 months and 47.6% after 3 to 7 years of follow-up [10].

CPAP is the most common method for the treatment of OSA, in which the patient wears a nasal mask that blows the air into the nostrils via a fan and keeps the airways open during sleep [8]. Research has shown that significant improvements in insomnia and snoring are feasible by using this device [7]. Most of the patients presenting with OSA begin their treatment with the CPAP device. The bi-level positive airway pressure (BiPAP) device is very similar to the CPAP machine. This device is often used for the treatment of central sleep apnea, a condition in which apnea occurs without obstruction of the respiratory tract. The BiPAP device may also be used if the patient is unable to endure CPAP therapy [10]. For the young patients who cannot tolerate a CPAP device, surgery is the best choice [10]. The patients should be hospitalized for a couple of days after the surgery and may have complications after soft palatal tissue reduction, such as nasal regurgitation of liquids [10]. Recently, laser-assisted uvulopalatoplasty (LAUP) has become more popular than UPPP [9]. OSA increases the risk of driving accidents and related mortality [11]. Patients with cerebral palsy are at increased risk of OSA. This condition is primarily managed by medication; however, tracheostomy is required to secure the airways in most cases [12]. In addition to the problems mentioned above, OSA is associated with pulmonary hypertension, right-sided heart failure, nocturnal hypoxemia, cerebrovascular accidents (CVA), cardiac arrhythmia and systemic hypertension [13].

Although OSA is a fairly common condition, it often remains undiagnosed. Because of the importance of the disease and its impact on morbidity and mortality of patients, family physicians should be familiar with the symptoms and treatments [11]. These patients can be treated by four surgical methods:

1- Nasal surgery (septoplasty, turbinate reduction, polyp removal, and sinus surgery)
2- Upper airway surgery (tonsillectomy, adenoidectomy, UPPP, and somnoplasty).
3- Lower airway surgery (genioglossus advancement, hyoid advancement, midline glossectomy, lingual tonsillectomy, bimaxillary advancement and tongue suspension suturing).
4- Obstruction-bypass surgery (tracheostomy) [7,11–14]. Patients with refractory epilepsy pose great clinical difficulties, such as a higher incidence of cognitive and psychological disorders, growth-plate injuries, mortality, and social stigmatization and isolation [15–17]. Neurosurgical techniques that involve specific areas of the cerebrum such as corpus callosotomy, temporal lobectomy, hemispherectomy and focal cortical resection may provide some reduction in seizure activity [16]. 60–80% of the epileptic patients are cured after anterior temporal lobectomy [8]. This is the only epilepsy surgery that is associated with fortuitous controlled tests and provides the best preservation against lateral temporal seizure attacks [18]. However, these techniques are effective in a limited number of patients. In addition, these invasive surgical methods cause postoperative complications, which reduce the number of suitable candidates for this type of treatment [19].

## CASE REPORT

**Fig. 1:**
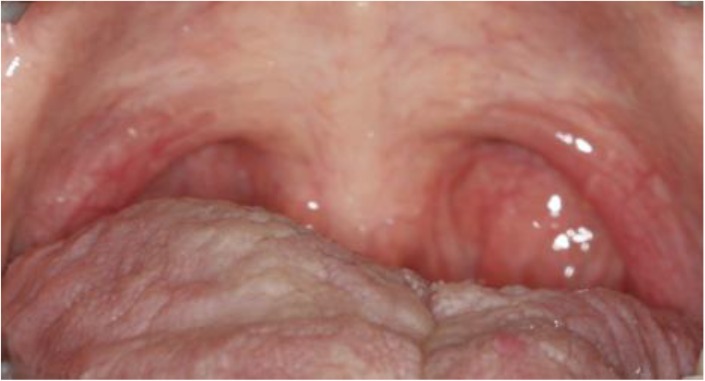
Preoperative intraoral view of the soft palate

**Fig. 2:**
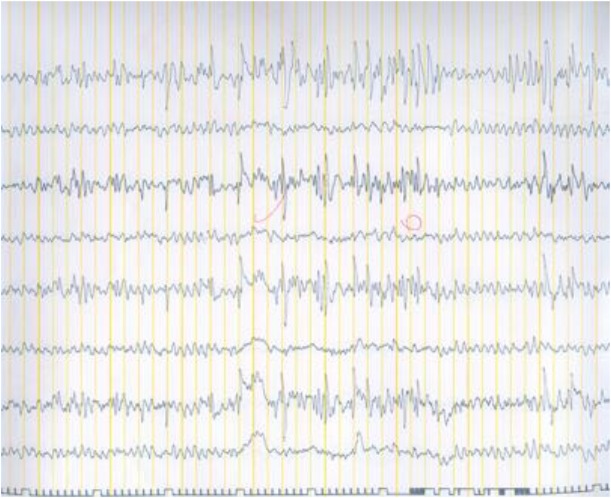
Preoperative EEG showing focal dysrhythmia and scattered sharp waves during hyperventilation

In December 2006, a 31-year-old woman was referred to a neurologist because of consciousness disorder and fainting. Her main problems were obesity, snoring and waking up with a feeling of suffocation in the middle of sleep. The intraoral examination showed a large soft palate (Fig. 1). The soft palate was scored as class III according to the Mallampati classification (visualization of the soft palate and the base of the uvula) [20]. The electroencephalogram (EEG) showed focal dysrhythmia during hyperventilation with scattered sharp waves (Fig. 2). The patient was depressed and had sleep disorders such as sleep apnea and myoclonus, especially at the onset of sleep. She had experienced several occurrences of complete loss of consciousness during swimming and at work. The patient was on anticonvulsants and antidepressants (at first, she had been prescribed with *Lamotrigine* for 5 months, but later she was given 500mg *Sodium valproate* per day).

One of the best treatments for snoring during sleep is UUUP. The success rate of this type of surgery is reported to be between 16% and 83% [21].

### Treatment procedure

We chose a minimally invasive surgical procedure for the present case since the patient had a proper facial profile and a large soft palate (class III according to the Mallampati classification) [20].

**Fig. 3:**
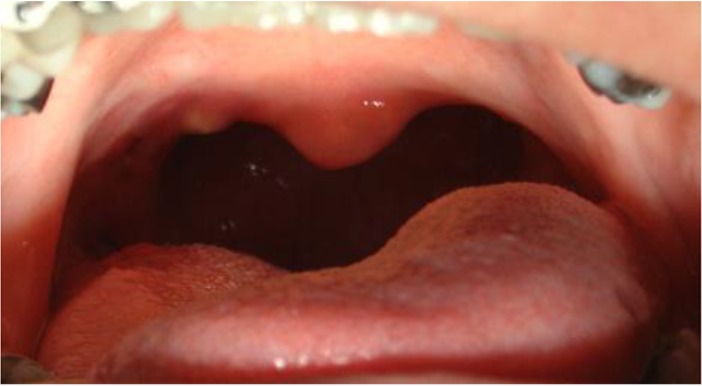
Postoperative intraoral view of the soft palate

In May 2007, after analyzing the lateral cephalogram, we evaluated the craniofacial and pharyngeal airway morphology before the surgery. Under general anesthesia, 1cm of the soft palatal mucosa, from the right tonsil to the left tonsil, was removed. The patient’s tonsils were also removed during the surgery, and the anterior and posterior tonsillar pillars were sutured together (Fig. 3).

**Fig. 4:**
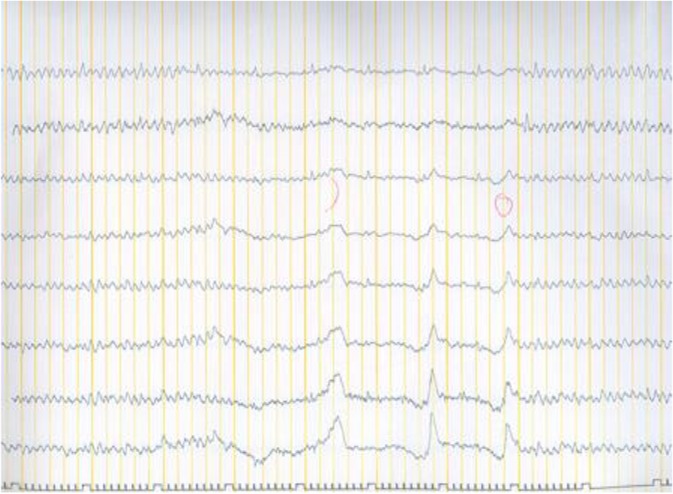
Postoperative EEG showing sharp waves at the level of the trachea

The symptoms were significantly decreased after the recovery. The patient no longer had sleep apnea, and antidepressants and antiepileptic drugs were discontinued. After the surgery, sharp waves were detected on the EEG at the level of the trachea (Fig. 4), but the patient was clinically asymptomatic. The 10-year follow-up showed no symptoms of sleep apnea or seizure. The patient did not lose any weight during the follow-up period.

## DISCUSSION

Sleep disorders can exacerbate the seizures in epileptic patients [22]. Dominici et al [23] stated that treating the sleep apnea will also decrease the frequency of epileptic seizures. Sleep fragmentation caused by respiratory disorders induces daytime sleepiness, migraine and attention deficits. In epileptic patients, apnea may facilitate seizures [23]. OSA occurs as the result of frequent narrowing of the upper airways during sleep. The patients are often overweight and have peripharyngeal fat [16] and enlarged tongue and soft palate [17]. Also, OSA occurs in some patients because of the problems associated with the jaws, such as micrognathia or retrognathia, which decrease the tongue space. These anatomical abnormalities reduce the cross-sectional area of the upper airway [14]. Reducing the tonicity of the airway muscles and increasing the gravitational pull in the supine position facilitate the normal air flow during breathing [14]. Partial obstruction of the airways may result in snoring, and there is a possibility of a complete obstruction in the supine position. A partial obstruction (hypopnea) or a complete apnea awaken the patient from sleep.

Usually, most of these arousals are minor and may happen several times during the night without being realized by the patient [6]. Soon after the patient falls back to sleep, the soft palate and tongue are again at rest and predisposed to snoring. People with chronic diseases are at increased risk of sleep disorders, as many patients with multiple sclerosis (MS) have reported symptoms of sleep disorders, which have affected their lifestyle [24]. Some studies have indicated a greater prevalence of sleep disorders in patients with spinal cord disease [25].

The sleep-wake cycle in patients presenting with OSA may be repeated several times during the night. Multiple awakenings and fragmented sleep cause daytime sleepiness in the patients, and they often complain of a disturbed sleep at night and drowsiness in the morning [14]. Many patients with OSA have facial abnormalities such as retrognathia, maxillary or mandibular hypoplasia and macroglossia; however, these symptoms may not be visible in some patients [14].

Orthognathic surgery is an invasive procedure used to remedy OSA. Patients with severe jaw problems and those who have not responded well to routine treatments are suitable candidates for this type of therapy [26].

Tracheostomy is a useful treatment for patients with a severe refractory OSA, although it is rarely performed today due to imposing several complications [8].

Preliminary studies have shown that treatment of OSA also improves some of the neurological problems [27]. Some authors have suggested that treatment of OSA can lead to adaptive changes in the neural structures that decrease sleepiness [28]. In general, studies have shown that a one-month CPAP treatment can improve the subjective memory [27,28]. Malow et al [29] reported that treatment of OSA had a positive effect on controlling epilepsy in 35 adults. Nath Zallek and Chervin [30] stated that treating the OSA can lead to a reduction in the frequency of cluster headaches.

## CONCLUSION

Since epilepsy and OSA may be correlated, it is extremely important to rule out sleep apnea in epileptic patients. Treatment of OSA, with the aim of reducing the frequency of seizures, requires collaborative studies between maxillofacial and neurological rehabilitation teams to get the best treatment results.
